# Post-operative Venous Thromboembolism Prophylaxis for Non-arthroplasty Knee Surgery: A Closed Loop Clinical Audit

**DOI:** 10.7759/cureus.89461

**Published:** 2025-08-06

**Authors:** Hassan Imtiaz, Emmanuel Anaga, Georgios Kouklidis, Majella Horgan

**Affiliations:** 1 Trauma and Orthopaedics, University Hospitals Dorset, Poole, GBR; 2 Orthopaedics, Royal National Orthopaedic Hospital, London, GBR; 3 Orthopaedics, University Hospitals Dorset, Poole, GBR; 4 Spinal Surgery, Royal National Orthopaedic Hospital, London, GBR

**Keywords:** arthroscopy, elective orthopaedics, knee surgery, non-arthroplasty, venous thromboembolism prophylaxis

## Abstract

Background: Venous thromboembolism (VTE) is a preventable complication following orthopaedic surgery. While most guidelines focus on arthroplasty, a significant number of knee surgeries fall under non-arthroplasty procedures, where post-operative VTE prophylaxis recommendations vary depending on anaesthetic time and weight-bearing status. National guidelines and available literature suggest the use of VTE prophylaxis for these cases, yet adherence in clinical practice remains inconsistent. To evaluate and improve compliance with NICE (National Institute for Health and Care Excellence) guidelines on VTE prophylaxis following non-arthroplasty knee surgery at a tertiary orthopaedic centre.

Methods: A closed-loop audit was conducted. Patient data from two six-month cycles (January-June 2023 and July-December 2023) were retrospectively reviewed. Inclusion criteria were patients aged ≥18 years undergoing non-arthroplasty knee procedures under the joint replacement unit. Compliance with NICE standards was assessed, and outcomes were compared pre- and post-intervention, which included a teaching session for the surgical team.

Results: In the first cycle (n=46), compliance with VTE prophylaxis was 50% for arthroscopic cases with anaesthetic time >90 minutes, and 36% for other non-arthroplasty surgeries. Post-intervention (n=66), compliance improved to 55% and 68% respectively. The 5% improvement in arthroscopic procedures was not statistically significant (p=1.0), whereas the 32% improvement in other non-arthroplasty procedures was statistically significant (p=0.02).

Conclusion: The audit identified significant gaps in adherence to VTE prophylaxis guidelines for non-arthroplasty knee procedures. Targeted education led to a notable improvement in compliance, particularly in non-arthroscopic cases. Sustained efforts, including documentation standardisation and ongoing training, are essential to maintain and further improve prophylactic practices.

## Introduction

Venous thromboembolism (VTE) refers to the development of a blood clot in a vein, resulting in partial or complete obstruction of blood flow [1]. This is a common complication following orthopaedic surgery and can be easily prevented if appropriate prophylaxis is administered after surgery [2].

The surgical workload for procedures of the knee comprises primarily joint arthroplasty, but there are a significant number of procedures that do not involve joint replacements [3-6], and can broadly be termed as non-arthroplasty surgeries of the knee. This latter cohort of patients can have a varying post-operative weight-bearing status and duration. Therefore, the recommended mode and duration of VTE prophylaxis are quite variable. Studies indicate that although the highest incidence of post-operative VTE events in orthopaedic surgery is linked to joint replacements, there is still a risk of this complication for non-arthroplasty knee procedures [7,8].

NICE (National Institute for Health and Care Excellence) guidelines, which form the foundations of working standards in the National Health Service across the United Kingdom, have been published to set out the standards regarding choice of post-operative VTE prophylaxis in non-arthroplasty knee surgeries, recommending VTE prophylaxis in case of an anaesthetic time greater than 90 minutes for arthroscopic procedures and for all those undergoing other non-arthroplasty knee procedures whose risk of VTE outweighs the bleeding risk [9]. Keeping this in mind, we aimed to evaluate the compliance with NICE guidelines at our trust with regards to prescribing appropriate VTE prophylaxis for non-arthroplasty knee procedures, and target optimal compliance following appropriate interventions.

## Materials and methods

A closed-loop audit cycle evaluating the compliance with VTE risk assessments done following non-arthroplasty knee procedures was conducted at a tertiary care orthopaedic hospital.

Data was collected retrospectively for both audit loops. The first loop looked at patient data between 01/01/2023 and 30/06/2023, while the second loop evaluated patients between 01/07/2023 and 31/12/2023. The standards for the audit were derived from National Institute for Health and Care Excellence (NICE) guidelines and are given in Table 1 [9]. 

**Table 1:** Audit Standards VTE: Venous thromboembolism

Audit Standard	Target Compliance (%)
Patients undergoing an arthroscopic procedure should receive VTE prophylaxis following the procedure if the anaesthetic time is 90 mins or more, or their VTE risk outweighs bleeding risk	100
All patients undergoing non-arthroplasty surgery other than arthroscopies should be given VTE prophylaxis post-procedure, or their VTE risk outweighs bleeding risk	100

The agreed target compliance was set at 100%. Patients over the age of 18 years, admitted under the joint replacement unit, undergoing elective non-arthroplasty surgery, met the inclusion criteria. Patients who were already on mechanical or chemical VTE prophylaxis, or those presenting with undergoing acute procedures for trauma, were excluded.

Data was collected from the Clinical Portal and NoteOn. Patient demographics, pre-operative VTE risk assessment, type of surgery, total anaesthetic time, and post-operative instructions, including weight-bearing status and VTE prescription, comprised the measured outcomes.

Following the findings of the first audit loop, a formal teaching session was conducted for the joint replacement unit team by a senior consultant, highlighting deficient areas and the need to clearly state anaesthetic time, weight-bearing status instructions and VTE prescription on operation notes and post-operative instructions. The second loop was conducted following this, in order to evaluate the effectiveness of these measures.

Data was collected and analysed on Microsoft Excel, with patient identifiers removed. Fischer’s Exact test was used to test for statistical significance. A p-value of <0.05 was used as a cut-off for statistical significance.

## Results

For the first loop of the audit, a total of 46 patients underwent non-arthroplasty surgery during the selected time period. Twenty-one out of forty-six (46%) of these were arthroscopic procedures, with four (19%) having anaesthetic time greater than 90 minutes. All of these patients were assessed to have one or more VTE risk factors on initial VTE risk assessment. Only 2/4 (50%) of these patients received low-molecular-weight heparin (LMWH)/aspirin following the procedure.

All patients undergoing arthroscopic procedures had full weight-bearing status after surgery. For the remaining 25/46 (54%) patients who underwent non-arthroplasty surgery, only 9/25 (36%) were prescribed chemical VTE prophylaxis. Among these, 5/9 had non-weight-bearing or partial weight-bearing status following surgery. Sixteen out of twenty-five (64%) patients in this group did not receive any form of VTE prophylaxis following surgery, with three having non-weight-bearing or partial weight-bearing status following surgery. The results for this first audit loop are given in Table 2.

**Table 2:** First Audit Loop Results

Procedure	Total Patients (n)	Anaesthetic Time (> 90 mins)	VTE Prophylaxis Prescribed (n)	Compliance (%)
Arthroscopy	21	4	2	50
Non-arthroplasty surgery	25	-	9	36

The compliance with prescribing appropriate VTE prophylaxis in patients undergoing arthroscopy procedures was 50% (2/4), while it was only 36% (9/25) for those who underwent other non-arthroplasty surgeries.

Following increased awareness through formal teaching sessions, the second loop was conducted, and 56 patients were evaluated during the specified time frame. Twenty-six of sixty-six (39%) were arthroscopic procedures, with 11/26 (42%) having an anaesthetic time greater than 90 mins. All of these patients were assessed to have more than one VTE risk factor. Only 6/11 (55%) of these patients received LMWH/Aspirin following the procedure. Twenty of twenty-six patients undergoing arthroscopic procedures had full weight-bearing status, while six patients had non- or partial weight-bearing after surgery. For the remaining 40/66 (61%) patients who underwent non-arthroplasty surgery, 27/40 (68%) were prescribed VTE prophylaxis (LMWH/aspirin). Among these, 15/27 (56%) had non-weight-bearing or partial weight-bearing status following surgery. Thirteen of forty (32%) patients in this group did not receive any form of VTE prophylaxis following surgery. One out of these had a non-weight-bearing or partial weight-bearing status following surgery.

The results of the second loop are given in Table 3. 

**Table 3:** Second Audit Loop Results VTE: Venous thromboembolism

Procedure	Total Patients (n)	Anaesthetic Time (> 90 mins)	VTE Prophylaxis Prescribed (n)	Compliance (%)
Arthroscopy	26	11	6	55
Non-arthroplasty surgery	40	-	27	68

None of the patients were reported to have developed a venous thromboembolic event post-procedure.

The audit demonstrated an improvement of 5% in terms of prescribing appropriate VTE prophylaxis for patients undergoing arthroscopic procedures (no statistical significance), while an improvement of 32% was observed in cases pertaining to other non-arthroplasty procedures (statistically significant, p=0.02). The results with statistical analysis are given in Table 4.

**Table 4:** Statistical Analysis *Fischer’s Exact test used for statistical significance (p<0.05 considered significant).

Arthroscopy	VTE Prophylaxis Given (n)	VTE not Prescribed (n)	Total Patients (n)	P-value*
First loop	2	2	4	-
Second loop	6	5	11	1.0
Non-arthroplasty	VTE prophylaxis given (n)	VTE not prescribed (n)	Total Patients (n)	P-value*
First loop	9	16	25	-
Second loop	27	13	40	0.02

## Discussion

This audit identified suboptimal prescribing practices of VTE prophylaxis in patients undergoing non-arthroplasty knee surgeries. Existing literature indicates a low to moderate risk of post-operative VTE in this patient population [10-12]. Nonetheless, national and international guidelines advocate the use of appropriate VTE prophylaxis - mechanical or chemical - to mitigate this preventable complication [2,13,14].

We believe that non-arthroplasty surgeries are often overlooked with regard to VTE risk assessment and appropriate VTE prophylaxis prescription because they are usually less invasive and shorter duration procedures compared to knee arthroplasties, with patients likely being discharged the same day as day cases. This has been highlighted by the audit results presented in this article.

While an improvement in VTE prophylaxis prescribing was observed during the second audit cycle following targeted educational interventions, optimal compliance was not achieved. Given that VTE events are largely preventable through appropriate prophylaxis, there is a clear need for more robust, sustained interventions. These may include routine staff education, enhanced clinical protocols, and systematic reminders integrated into clinical workflows.

We suggest the use of VTE checklists with discharge summaries, which contain tick boxes to confirm the duration of surgery, VTE risk assessment and the relevant criteria for non-arthroplasty surgeries as recognised through NICE guidelines [9]. With such checklists, it can be easier for the discharging nurse or doctor to quickly assess the need for VTE prophylaxis, therefore ensuring patient safety.

## Conclusions

This audit identified significant gaps in adherence to VTE prophylaxis guidelines following non-arthroplasty knee procedures. Targeted educational interventions resulted in improved compliance, particularly in non-arthroscopic cases, although gaps remained. Sustained efforts - including standardised documentation, checklist implementation, and ongoing multidisciplinary training - are essential to maintain and further enhance prophylactic practices and ensure optimal patient outcomes.
